# Determination of the Size Distribution of Metallic Colloids from Extinction Spectroscopy

**DOI:** 10.3390/nano11112872

**Published:** 2021-10-28

**Authors:** Yehia Mansour, Yann Battie, Aotmane En Naciri, Nouari Chaoui

**Affiliations:** Université de Lorraine, LCP-A2MC, F-57000 Metz, France; yehia.mansour@univ-lorraine.fr (Y.M.); aotmane.en-naciri@univ-lorraine.fr (A.E.N.); nouari.chaoui@univ-lorraine.fr (N.C.)

**Keywords:** colloids, nanoparticle size distribution, extinction spectroscopy, Mie theory

## Abstract

In this paper, we explore the ability of extinction spectroscopy to characterize colloidal suspensions of gold nanoparticles (Au NPs). We demonstrate that the Au NPs’ size distribution can be deduced by analyzing their extinction spectra using Mie theory. Our procedure, based on the non-negative least square algorithm, takes advantage of the high sensitivity of the plasmon band to the Au NP size. In addition, this procedure does not require any a priori information on the Au NP size distribution. The Au NPs’ size distribution of monomodal or bimodal suspensions can be satisfactorily determined from their extinction spectra. Finally, we show that this characterization tool is compatible with in situ measurement and allows following the change in NPs’ radii during laser exposure.

## 1. Introduction

Colloidal suspensions of noble metal nanoparticles (NPs) exhibit strong optical absorption due to surface plasmon resonance (SPR) of NPs. The position and width of the SPR bands depend on the NPs’ size, shape, and environment [1,2]. This tunability makes metallic NPs great candidates for a wide range of applications such as photothermal therapy [3], plasmonic catalyzers [4], chemical sensors [5], or optical filters [6]. Conventional synthesis routes unavoidably produce NPs with sizes distributions that induce drastic changes in the optical properties of colloids. These distributions are commonly determined by transmission electron microscopy (TEM). However, since TEM is a local characterization tool, the observed NPs may be minimally or not representative of the whole sample, and TEM is often time-consuming. Thus, the development of nonlocal characterization techniques is crucial to evaluate NPs’ size distribution after their synthesis.

Nonlocal techniques such as small-angle X-ray scattering (SAXS) [7,8,9], wide-angle X-ray diffraction [10], mass spectrometry [11], dynamic light scattering (DLS) [12,13], gravitational sedimentation [14,15], optical pulse scattering [16], and analytical ultracentrifugation analysis (AUA) [17] are widely used to determine NPs’ size distribution of colloids. As SAXS is sensitive to NPs’ a few nanometers in size, it has been used to investigate the evolution of the size of gold nanoparticles (Au NPs) during their growth [7,8]. DLS only measures the hydrodynamic radius of NPs. Therefore, the NPs’ sizes can be overestimated due to the presence of capping agent around the NPs, which strongly interacts with the surrounding liquid [12,13,18]. In addition, as the scattering cross-section of large NPs is higher than that of small NPs, DLS could also produce misleading results if NPs are not monodisperse. AUA, which consists of the determination of the sedimentation coefficient of NPs, is a powerful method to evaluate complex NP size distributions. However, as pointed out by Mahl et al., AUA tends to underestimate NP size due to the presence of capping agent around the NPs [18].

Extinction spectroscopy is a noninvasive optical characterization technique, which can be used to gain quantitative insights on NP morphology [19,20,21,22,23,24,25]. Haiss et al. [19] used the position of the plasmon band and the ratio between the absorbance at the plasmon band and the absorbance at 450 nm to estimate the mean diameter of gold NPs. However, their approach does not take into account the NP size distribution. As shown by Eustis et al. [25], the aspect ratio distribution of metallic nanorods can be estimated by fitting their extinction spectra with Gans theory. We recently improved this approach by analyzing the extinction spectra with shape-distributed effective-medium theory [20,21]. Several efforts have also been devoted to evaluating NP size from extinction spectroscopy [9,26,27,28,29]. The optical properties of isolated spherical nanoparticles are well-described by Mie theory [30]. Several authors used this theory to determine NPs’ mean radius and concentration from their extinction spectra [26]. However, they did not take into account the polydispersity of NPs. As shown by Amandola et al. [29], the NP concentration estimated by analyzing the extinction spectra of Au colloids with Mie–Gans theory, is barely affected by the size distribution of Au NPs. This result was confirmed by recent simulations performed with modified Maxwell–Garnett–Mie theory [31].

In this context, the NP size distribution is introduced in Mie theory. By solving the inverse problem using Mie theory, we unambiguously demonstrate that the size distribution of spherical Au NPs can be estimated from their extinction spectra. Contrary to several works, our method, based on a constrained non-negative least square algorithm [32], does not require any a priori information concerning NP size distribution. The unique assumption is that NPs have a spherical shape. The comparison between the distribution obtained by TEM and extinction spectroscopy reveals the reliability of our method. We also show that the determination of the size distribution is fast enough to be suitable for in situ measurements.

## 2. Materials and Methods

The investigated colloids were purchased from Sigma-Aldrich (Saint-Quentin-Fallavier, France). These colloids consist in spherical Au NPs in water. TEM images of Au NPs were recorded with a Technai CM200 microscope operating at 200 kV. The TEM samples were prepared by evaporating a drop of colloidal suspension on a copper TEM grid. Extinction measurements of Au NP suspensions were recorded using a Horiba Jobin-Yvon spectrophotometer equipped with a quartz cell with a 1 mm light-path.

Laser-induced fragmentation of Au NPs was also conducted. In this experiment, 2 mL of gold colloids, introduced in a 1 cm light-path quartz cell, was horizontally exposed to nanosecond laser pulses. The suspension was continuously stirred with a magnetic stirrer during the laser exposure. The laser pulses were delivered from a Nd-YAG laser (Continuum Surelite I, Evry, France) set to its fundamental wavelength (1064 nm). The pulse width, repetition rate, and laser fluence were 6 ns, 10 Hz, and 3.2 J·cm^−2^, respectively. Extinction spectra of the suspensions were recorded in situ during the laser exposure by using a homemade set-up [33]. The latter was mainly composed of a 200 W mercury–xenon arc lamp source (Newport) and a compact UV–visible spectrophotometer (AvaSpec-ULS2048L-USB2 Avantes, Apeldoorn, The Netherlands). The extinction spectra of the colloidal suspension were recorded in the range of 400–900 nm with an acquisition rate of 10 Hz.

## 3. Model

Colloidal suspensions are composed of spherical Au NPs in water. In the following, the presence of surfactant is neglected. The extinction cross-section of an isolated spherical NP with radius *R* calculated for a wavelength *λ* from Mie theory [30] is given by the following expression:

(1) $$\sigma_{ext}\left(R,\lambda \right)=\frac{2\pi }{k^{2}}\overset{\infty }{\underset{n=1}{\sum }}\left(2n+1\right)Re\left(a_{n}+b_{n}\right)$$

where *k* is the norm of the wavevector; $a_{n}$ et $b_{n}$ are the Mie coefficients defined by the following equations:

(2) $$a_{n}=\frac{m\psi_{n}\left(mx\right)\psi_{n}^{\prime }\left(x\right)-\psi_{n}\left(x\right)\psi_{n}^{\prime }\left(mx\right)}{m\psi_{n}\left(mx\right)\xi_{n}^{\prime }\left(x\right)-\xi_{n}\left(x\right)\psi_{n}^{\prime }\left(mx\right)}$$

(3) $$b_{n}=\frac{\psi_{n}\left(mx\right)\psi_{n}^{\prime }\left(x\right)-m\psi_{n}\left(x\right)\psi_{n}^{\prime }\left(mx\right)}{\psi_{n}\left(mx\right)\xi_{n}^{\prime }\left(x\right)-m\xi_{n}\left(x\right)\psi_{n}^{\prime }\left(mx\right)}$$

with $x=kR\sqrt{\varepsilon_{m}}$; $m=\sqrt{\varepsilon_{np}/\varepsilon_{m}}$; $\psi_{n}$ and $\xi_{n}$ are the *n*th-order Riccatti–Bessel functions; and $\varepsilon_{np}$ and $\varepsilon_{m}$ are the dielectric function of the NPs and the host matrix, respectively. As shown from ellipsometric measurement performed on colloids [34], the presence of capping agent and counter ions in a suspension can be neglected and the dielectric function of water can be used as the dielectric function of the matrix. The dielectric function of Au NPs is given by [31]:

(4) $$\varepsilon_{NP}=\varepsilon_{bulk}-\frac{\omega_{p}^{2}}{\omega \left(\omega +i\Gamma_{0}\right)}+\frac{\omega_{p}^{2}}{\omega \left(\omega +i\left(\Gamma_{0}+\frac{v_{f}}{R}\right)\right)}$$

where $\varepsilon_{bulk}$ is the dielectric function of bulk given by Johnson [35]; $\omega_{p}=8.44$ eV and $\Gamma_{0}=0.08$ eV are the plasma frequency and the damping energy of gold, respectively; and $v_{f}=1.4\times 10^{6}$ m·s^−1^ is the Fermi velocity of free electrons. The first and second terms of Equation (4) are related to the contribution of interband transitions of bound electrons of bulk gold. The last term is associated with the intraband transitions of conduction electrons of gold NPs. The intraband transitions are described by the Drude dispersion. This dispersion law is modified to consider the intrinsic confinement effect that occurs for NP radii smaller than the mean free path of conduction electrons.

According to our previous work, the concentration of NPs in a suspension is sufficiently small so the electromagnetic coupling between NPs can be neglected [34]. Therefore, the extinction coefficient of the suspension can be obtained by weighting the extinction cross-section of individual Au NPs by their concentration.

(5) $$\alpha \left(\lambda \right)=\overset{n}{\underset{i=1}{\sum }}N\left(R_{i}\right)\sigma_{ext}\left(R_{i}\right)$$

$\mathrm{where}\;N\left(R_{i}\right)$ is the concentration of NPs that have a radius $R_{i}$. In the following, we use a constant step size $\Delta R=R_{i+1}-R_{i}$. By using the same procedure regardless of the wavelength, we obtain a set of linear equations that can be rewritten as follows:

(6) Y=AX

with

$$Y=\left(\begin{array}{c} \alpha \left(\lambda_{1}\right)\\ \vdots \\ \alpha \left(\lambda_{m}\right) \end{array}\right),\;A=\left(\begin{array}{ccc} \sigma_{ext}\left(\lambda_{1},R_{1}\right) & \cdots & \sigma_{ext}\left(\lambda_{1},R_{n}\right)\\ \vdots & \ddots & \vdots \\ \sigma_{ext}\left(\lambda_{m},R_{1}\right) & \cdots & \sigma_{ext}\left(\lambda_{m},R_{n}\right) \end{array}\right)\;\mathrm{and}\;X=\left(\begin{array}{c} N\left(R_{1}\right)\\ \vdots \\ N\left(R_{n}\right) \end{array}\right)$$

The vectors *Y* and *X* are related to the extinction spectra and the NP size distribution of colloids, respectively. The matrix *A* is calculated by using Equation (1). For a given distribution of NP size, Equation (6) allows a simulation of the extinction spectra of the colloidal suspension. Equation (6) can also be used to estimate the NPs’ size distribution from their extinction spectra. The mathematical solution is given by the following ordinary least square regression:

(7) $$X={\left(A^{T}A\right)}^{-1}A^{T}Y$$

The mathematical solution given by Equation (7) can be unphysical because this procedure does not ensure positive concentrations. To avoid unphysical solutions, we decided to solve the following constrained non-negative least square problem:

(8) $$X=\arg{min}_{X}\left(AX-Y\right)\;\mathrm{with}\;N\left(R_{i}\right)>0$$

The vector of NP size distribution (*X*) is obtained without prior information concerning the NPs’ size distribution by using the non-negative least square algorithm [32]. The choice of the step size ΔR in Equations (7) and (8) has a significant impact on the estimated size distribution. To estimate the optimal value of radius step, we introduce two parameters, *e*_1_ and *e*_2_, defined as follows:

(9) $$e_{1}=\|Y_{mes}-AX\|$$

(10) $$e_{2}=\|\left(A^{T}A\right){\left(A^{T}A\right)}^{-1}\|/\sqrt{n}$$

**Figure 1:**
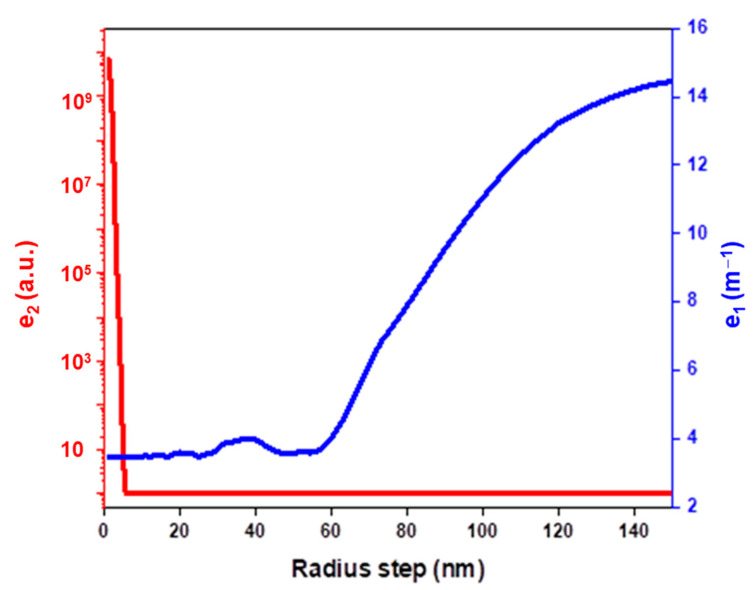
Evolution of *e*_1_ and *e*_2_ with radius step.

*e*_1_ traduces the difference between the measured spectra ($Y_{mes}$) and the modeled spectra (AX). It describes the ability of our model to reproduce spectroscopic measurements. *e*_2_ was used to evaluate the numerical accuracy of the matrix inversion ${\left(A^{T}A\right)}^{-1}$ which appears in Equation (7). *e*_2_ is equal to 1 for an invertible matrix, whereas it tends toward infinity for a close-to-singular matrix. Figure 1 shows the evolution of *e*_1_ and *e*_2_ with radius step.

*e*_1_ is almost constant for a ΔR smaller than 60 nm, whereas it drastically increases for higher radius step. In other words, the spectra cannot be reproduced for a too-large sampling step size. *e*_2_ is equal to 1 for step size higher than 4 nm. However, it diverges for smaller step size. Thus, for a too-small step size, the matrix A is ill-conditioned (i.e., despite the matrix being able to be inverted, it is close to a noninvertible matrix) and several distributions could produce the same optical signature. According to these results, we used, in the following, a step size of 5 nm. This step size was found to be sufficient to induce measurable changes in extinction spectra.

## 4. Results and Discussion

### 4.1. Simulations

**Figure 2:**
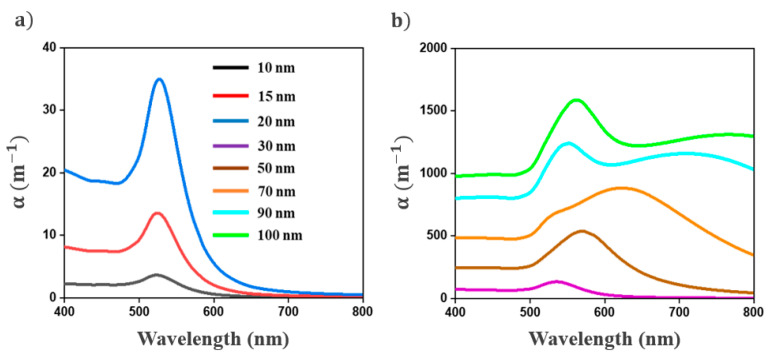
(**a**,**b**) Evolution of the extinction spectra of gold colloids with NP radius. The concentration of NPs was set to 10^20^ m^−3^.

The influence of NP size on the extinction spectra of colloids is illustrated in Figure 2 through some simulations based on Mie theory (Equation (6)). These simulations were performed by considering all Au NPs in the suspension as having the same radius. The concentration of NPs was set to 10^20^ m^−3^.

Figure 2 shows that the amplitude of the extinction spectra increases with NP radius. According to the Rayleigh approximation, the absorption and the scattering cross-sections of NPs are proportional to the volume and the square of the volume of the NPs, respectively. In the 400–500 nm spectral range, the extinction coefficient is almost independent of the wavelength and is mainly dominated by the interband transitions of Au NPs. The plasmonic effects can only be observed at wavelengths above 500 nm. For a NP radius smaller than 30 nm, the extinction spectra exhibit a band centered at 525 nm, attributed to the dipolar plasmon mode of NPs. This plasmon band is progressively redshifted and broadened for a mean NP radius larger than 20 nm. These spectral variations are attributed to dynamic effects and radiation damping, which are due to the nonuniformity of the electric field inside large NPs [2,31]. For a NP radius larger than 70 nm, a second band can be observed [2]. This band, assigned to the quadrupolar plasmon mode of NPs, is progressively redshifted from 530 to 565 nm as the NP radius increases from 70 to 100 nm. These results confirm that the extinction spectra are sensitive to NP radius.

**Figure 3:**
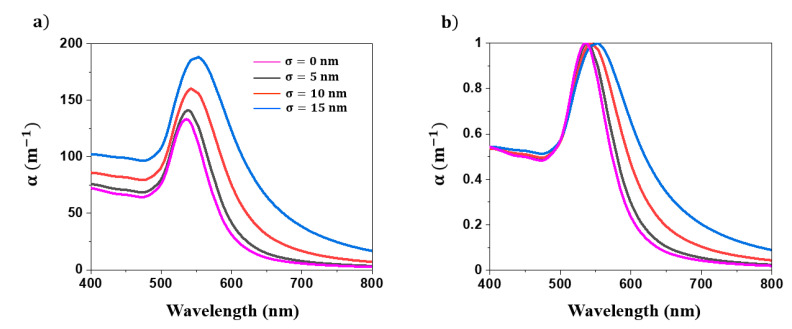
(**a**) Influence of the standard deviation (σ) of the Au NPs size distribution on their extinction spectra. (**b**) Normalized extinction spectra of Au NPs. These simulations are performed by considering a Gaussian distribution centered at 30 nm. The concentration of NPs is set to 10^20^ m^−3^_._

For the purpose of evaluating the impact of the polydispersity of NPs, the extinction spectra of colloids were calculated by considering a Gaussian NP size distribution with various standard deviations. The NPs’ mean radius and concentration were set to 30 nm and 10^20^ m^−3^, respectively. As shown in Figure 3, as the standard deviation (σ) of the NPs size distribution increases, the plasmon band broadens. The contribution of the larger NPs is traduced by an asymmetrical broadening of the plasmon band toward the red region of the spectra. Therefore, the symmetry of the plasmon band could provide insights into the polydispersity of the NPs’ population. Note that this broadening is accompanied by an increase in the plasmon band and a redshift in its position. When compared to the spectra in Figure 2, the plasmon band position appears to be more sensitive to the NPs’ mean radius than the polydispersity.

### 4.2. Characterization of Colloids

**Figure 4:**
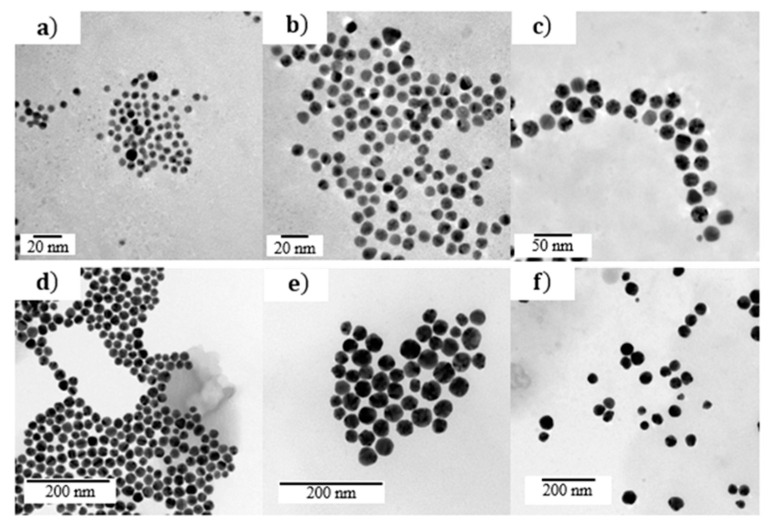
TEM images of (**a**) S1, (**b**) S2, (**c**) S3, (**d**) S4, (**e**) S5, and (**f**) S6 Au NPs.

**Figure 5:**
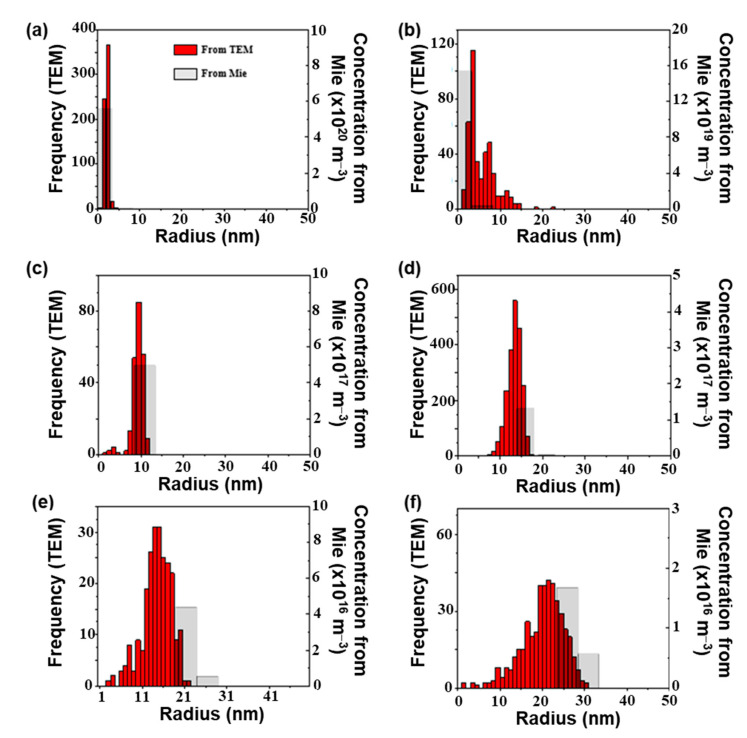
Comparison between the size distributions determined by TEM (in red) and extinction spectroscopy (in grey) of (**a**) S1, (**b**) S2, (**c**) S3, (**d**) S4, (**e**) S5, and (**f**) S6 Au NPs.

In order to confirm the agreement of the model with the experimental results, the measured extinction spectra of a gold colloidal suspension were compared to the calculated spectra. The extinction spectra of six commercial colloidal suspensions (Sigma Aldrich) denoted S_i_ (with i = 1, …, 6), were recorded in the spectral range of 400–800 nm and their NPs population was characterized by transmission electronic microscopy. As shown in Figure 4, these suspensions were composed of nearly spherical Au NPs in water. Their radius distributions, determined from TEM images processing, are shown in Figure 5. The mean radius of S_1_, S_2_, S_3_, S_4_, S_5_, and S_6_ NPs are 2, 4, 10, 14, 15, and 22 nm, respectively, and their polydispersity indexes, defined as the ratio between the standard deviation and the mean value of NPs radius, are 0.2, 0.3, 0.3, 0.1, 0.2, and 0.2, respectively.

**Figure 6:**
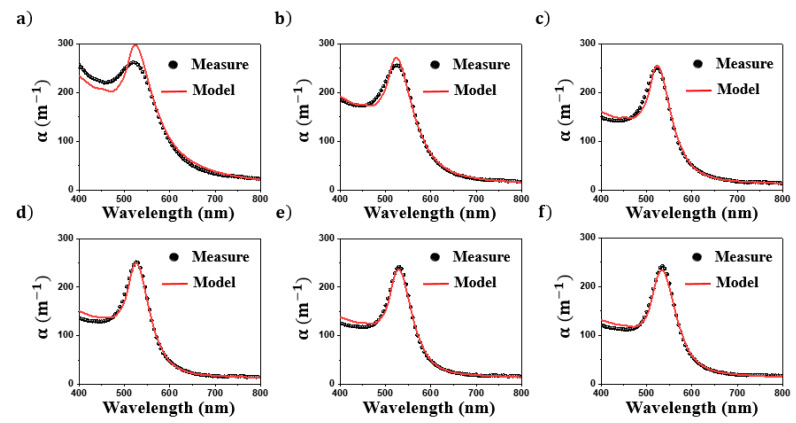
Comparison between the measured and modeled extinction spectra of (**a**) S1, (**b**) S2, (**c**) S3, (**d**) S4, (**e**) S5, and (**f**) S6 Au colloids.

The main feature of extinction spectra of these colloids, shown in Figure 6, is a single band that progressively shifts from 520 to 535 nm as the NPs’ mean radius increases. According to the simulations depicted in Figure 2 and the NP size determined by TEM (Figure 5), this band can be assigned to the dipolar plasmon mode of Au NPs. The intrinsic confinement effect occurring for small NPs explains the broadness of the plasmon band observed for S1. These results illustrate the correlation between the extinction spectra and the NPs’ size distribution.

The NP size distributions estimated by analyzing each spectrum with Equation (8) are presented in Figure 5. The NP sizes obtained from extinction spectroscopy are globally in agreement with those determined by TEM. The modes of the calculated distribution (Figure 5) satisfactorily predict those measured by TEM image processing. In addition, as shown in Figure 6, the extinction spectra calculated from these distributions are close to the measured ones. These results suggest that the distributions calculated from extinction spectroscopy have the same optical signature as the NPs population in the suspension. The deviation between the distribution obtained by TEM and extinction spectroscopy can be due to the optical constant of gold introduced in Mie theory, the small number of NPs measured by TEM, and the deviation of NP shape from perfect sphere. Several Au dielectric functions with large differences were reported in the literature [36]. Contrary to TEM, the number of NPs probed by extinction spectroscopy is estimated as 10^10^–10^12^ (depending on the colloidal suspension), which is sufficiently significant to provide an accurate estimation of the NPs’ size distribution. Concerning the shape of NPs, we found, by analyzing the extinction spectra with shape-distributed effective-medium theory [20,21] (not shown), that NPs can be considered spherical. Some improvements beyond the scope of this study can be proposed. First, the dielectric function of gold NPs could be estimated on benchmark colloids. Second, Mie theory could be replaced by other numerical approaches, such as the boundary element method or discrete dipole approximation, to calculate the extinction cross-section of nonspherical NPs.

**Figure 7:**
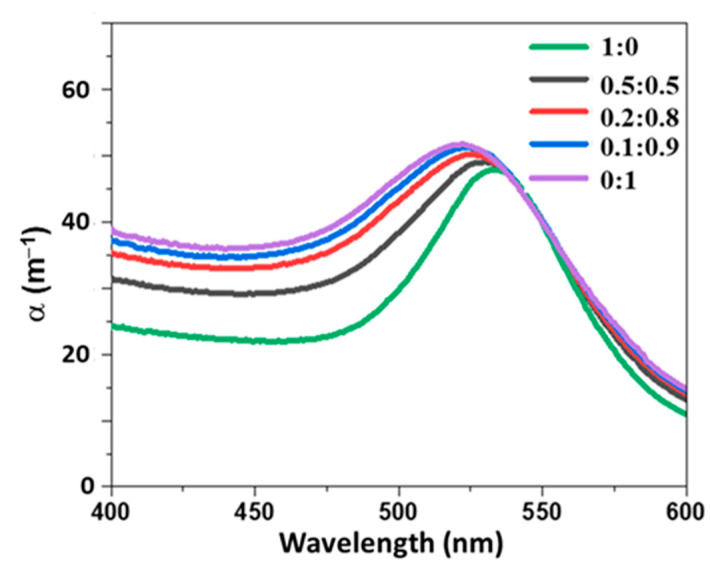
Measured extinction spectra of mixtures of P1 and P2 suspensions. Five volume ratios of P1 to P2 are considered.

**Figure 8:**
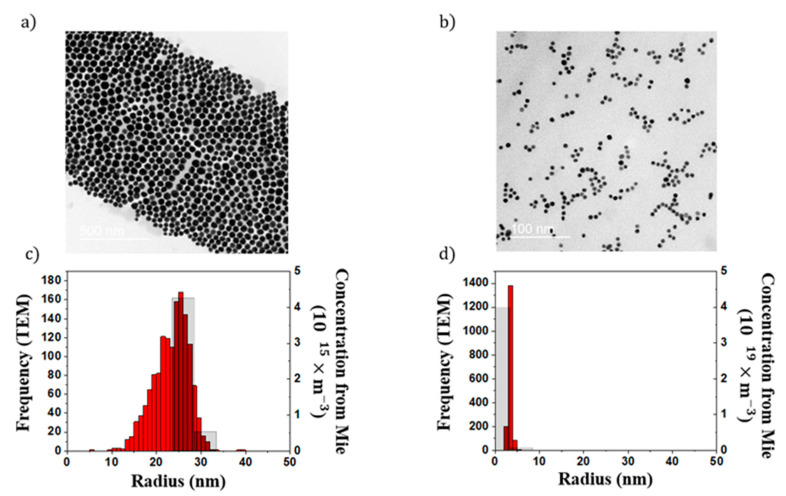
(**a**,**b**) TEM images of (**a**) P1 and (**b**) P2 NPs. (**c**,**d**) NPs size distribution of (**c**) P1 and (**d**) P2 determined by TEM (in red) extinction spectroscopy (in grey).

**Figure 9:**
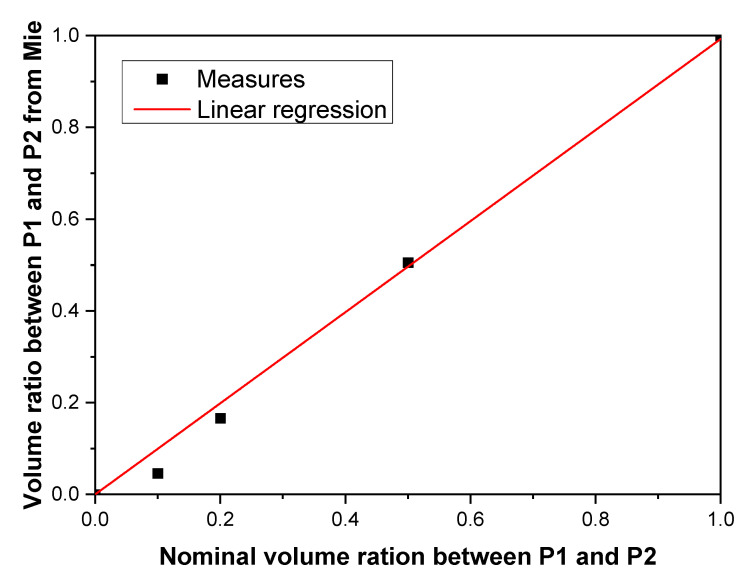
Comparison between the volume ratio of P1 and P2 suspensions determined from extinction spectroscopy and the nominal volume ratio. The linear regression is depicted as a red line. The slope of the linear regression is 0.99 ± 0.22.

The presence of a bimodal distribution of NPs is a crucial issue for several characterization tools such as DLS. To investigate the robustness of our method, we produced three suspensions by mixing two colloidal suspensions (P1 and P2) at several volume ratios (0.5:0.5, 0.2:0.8, and 0.1:0.9). The extinction spectra of these suspensions, measured in the 400–600 nm spectral range, are shown in Figure 7. The spectra of the P1 and P2 suspensions exhibit a plasmon band centered at 533 and 521 nm, respectively. The spectra of P2 shows a broader plasmon band than P1. The spectra of the mixture lies between the spectra of P1 and P2. In addition, the plasmon band is progressively blueshifted as the volume ratio between P2 and P1 increases, suggesting that the extinction spectra are sensitive to this parameter. The NP size distributions of P1 and P2, extracted from extinction spectra, are reported in Figure 8. P1 was mainly composed of NPs with a radius in the 23–33 nm range; those constituting P2 had a radius smaller than 8 nm. These sizes are in agreement with TEM measurements, which described a mean radius of 24 and 4 nm, for P1 and P2, respectively (Figure 8). The distribution of the mixture (not shown) deduced from the extinction spectra are composed of the same population as P1 and P2 NPs. The difference between mixtures was due to the relative amount of both populations of NPs. The volume ratio between both suspensions can be directly determined from the relative concentration of P1 and P2 populations of NPs estimated by extinction spectroscopy. As shown in Figure 9, the relative amount between both populations of NPs determined by extinction spectroscopy increases linearly with the nominal volume ratio between P1 and P2, with a slope estimated as 1.03. According to these results, the two modes of bimodal populations of NPs can be distinguished using extinction spectroscopy. In other words, contrary to DLS, the high sensitivity of the plasmon band to the NP size can be used to gain quantitative insights on the relative concentration of both populations of NPs.

**Figure 10:**
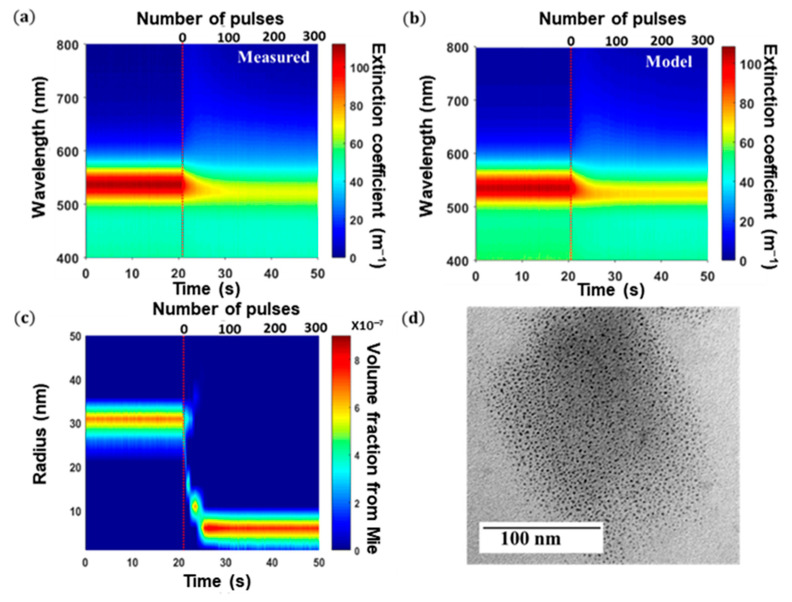
Evolution of (**a**) the measured and (**b**) modeled extinction spectra of Au colloids during laser exposure. (**c**) Evolution of the NP size determined from extinction spectroscopy during laser exposure. The red dashed line shows the beginning of laser exposure. The radius step is DR = 5 nm. An interpolation was used to plot the data. (**d**) TEM image of S6 NPs after 30 min laser exposure. The laser fluence, wavelength, and repetition rate were 3.2 J·cm^−1^, 1064 nm, and 10 Hz, respectively.

In order to evaluate the potential of our technique for in situ characterization, we recorded the extinction spectra of the diluted S6 sample during its exposure to Nd:YAG laser pulses. As shown in Figure 10a, the extinction spectra of the suspension, measured with an acquisition rate of 10 Hz, drastically changed during the exposure. The plasmon band, initially centered at 535 nm, progressively blueshifted to 523 nm. This variation was accompanied by a decrease in the width and the magnitude of the plasmon band. Each recorded extinction spectrum was then analyzed with our model. As shown in Figure 10b, the model globally reproduces the measured extinction spectra. Figure 10c reports the evolution of the NPs’ size distribution during laser exposure. The NP size decreased from 25–35 to 1–7 nm after 6 s of laser exposure. The distribution estimated at the final state is in accordance with those measured by TEM (Figure 10d). As reported by several works, this NP size reduction is induced by the fragmentation of NPs that occurs during laser exposure [37]. The absorption of a laser pulse by the NPs induces a rise in its temperature until it reaches the boiling temperature of gold. Then, small NPs growth via a coalescence mechanism from the vaporized gold atoms. The advantage of our technique is that the matrix A of Equation (6) is calculated only once, before the measurements. This matrix is then reused to analyze all spectra in real time. The central processing unit (CPU) time spent to analyze one spectra is 50 ms. This example reveals that in situ extinction spectroscopy coupled with our model can be used to investigate the kinetics of NP size modification.

We now provide a nonexhaustive comparison between our approach and standard characterization tools. Contrary to local characterization techniques such as TEM, the distribution deduced from Mie theory is obtained on a large number of NPs. However, Mie theory is only valid for spherical NPs, while TEM measurements can be performed on nonspherical NPs. In addition, the radius step size used in our approach is much larger than those determined by TEM. Contrary to measurements based on the diffusion of NPs such as DLS, our approach directly describes the NPs radius. DLS which is sensitive to the hydrodynamic radius of NPs, tends to overestimate NP size. Finally, contrary to techniques based on X-ray scattering, our approach does not require large-scale facilities and can easily be used in line to control the production of colloids.

## 5. Conclusions

In summary, we introduced NP size distribution in Mie theory. Simulations performed with this theory confirmed that the position and width of the plasmon band of Au NPs strongly depend on the NPs size distribution. We showed that the NPs’ size distribution can be determined by analyzing extinction spectra measured on Au colloids with Mie theory without any a priori information concerning size distribution. The unique assumption is that NPs are perfectly spherical. Contrary to DLS or TEM measurements, our technique allows characterizing bimodal distributions and is suitable for in situ characterization. As extinction spectroscopy is a nonlocal technique, the NPs’ size distribution was evaluated on a large number of NPs estimated as 10^10^–10^12^. As a proof of concept, we decided to focus our investigation on spherical gold NPs. This method can also be applied to other plasmonic nanoparticles such as silver or copper nanoparticles. The plasmon band of plasmonic nanoparticles is sensitive to nanoparticle size. Our approach can be easily extended to other NP shapes by calculating matrix A with other formalisms such as the boundary element method or discrete dipole approximation. However, this extension is beyond the scope of this study and will be reported in other publications.

## Data Availability

The data presented in this study are available on request from the corresponding author.
